# The Effects of Climate Change and Globalization on Mosquito Vectors: Evidence from Jeju Island, South Korea on the Potential for Asian Tiger Mosquito (*Aedes albopictus*) Influxes and Survival from Vietnam Rather Than Japan

**DOI:** 10.1371/journal.pone.0068512

**Published:** 2013-07-24

**Authors:** Su Hyun Lee, Kwang Woo Nam, Ji Yeon Jeong, Seung Jin Yoo, Young-Sang Koh, Seogjae Lee, Sang Taek Heo, Seung-Yong Seong, Keun Hwa Lee

**Affiliations:** 1 Jeju National University School of Medicine, Jeju, South Korea; 2 Wide River Institute of Immunology, Seoul National University College of Medicine, Daehakno, Seoul, South Korea; 3 Department of Microbiology and Immunology, Department of Biomedical Sciences, Seoul National University College of Medicine, Daehakno, Seoul, South Korea; Thomas Jefferson University, United States of America

## Abstract

**Background:**

Climate change affects the survival and transmission of arthropod vectors as well as the development rates of vector-borne pathogens. Increased international travel is also an important factor in the spread of vector-borne diseases (VBDs) such as dengue, West Nile, yellow fever, chikungunya, and malaria. Dengue is the most important vector-borne viral disease. An estimated 2.5 billion people are at risk of infection in the world and there are approximately 50 million dengue infections and an estimated 500,000 individuals are hospitalized with dengue haemorrhagic fever annually. The Asian tiger mosquito (*Aedes albopictus*) is one of the vectors of dengue virus, and populations already exist on Jeju Island, South Korea. Currently, colder winter temperatures kill off Asian tiger mosquito populations and there is no evidence of the mosquitos being vectors for the dengue virus in this location. However, dengue virus-bearing mosquito vectors can inflow to Jeju Island from endemic area such as Vietnam by increased international travel, and this mosquito vector's survival during colder winter months will likely occur due to the effects of climate change.

**Methods and Results:**

In this section, we show the geographical distribution of medically important mosquito vectors such as *Ae. albopictus*, a vector of both dengue and chikungunya viruses; *Culex pipiens*, a vector of West Nile virus; and *Anopheles sinensis*, a vector of *Plasmodium vivax*, within Jeju Island, South Korea. We found a significant association between the mean temperature, amount of precipitation, and density of mosquitoes. The phylogenetic analyses show that an *Ae. albopictus*, collected in southern area of Jeju Island, was identical to specimens found in Ho Chi Minh, Vietnam, and not Nagasaki, Japan.

**Conclusion:**

Our results suggest that mosquito vectors or virus-bearing vectors can transmit from epidemic regions of Southeast Asia to Jeju Island and can survive during colder winter months. Therefore, Jeju Island is no longer safe from vector borne diseases (VBDs) due to the effects of globalization and climate change, and we should immediately monitor regional climate change to identify newly emerging VBDs.

## Introduction

Global mean air temperatures have risen at a faster rate than at any time since records began to be kept in the 1850s, and temperatures are expected to increase by another 1.8 to 5.8°C by the end of this century [1]. Climate change is expected to have enormous implications for human health, especially due to of vector-borne diseases (VBDs) [1]. VBDs are infections transmitted by the bite of infected arthropod species such as mosquitoes, ticks, triatomine bugs, sandflies, and blackflies [2]. VBDs are among the most well studied climate change related diseases because insect vectors tend to be more active at higher temperatures [1]. Numerous studies showed that high temperature and precipitation can lead to an increase in disease transmission and there is some evidence of climate change-related shifts in the distribution of tick vectors of disease, some non-malarial mosquito vectors in Europe and North America, and in the phenology of bird reservoirs of pathogens [2], [3]. Prior research also showed that the development rates of vector-borne pathogens such as dengue virus, West Nile virus, yellow fever virus, chikungunya virus, and malaria parasites are strongly temperature dependent [4].

This pattern may also be occurring in parts of South Korea due to the effects of climate change. Jeju Island is located at the southern end of the Korean Peninsula, which is the hottest area and is classified as a subtropical weather zone [5]. Thus, the island would be more affected by climate change than would other regions of South Korea. Jeju Island has a temperature gradient now exists between the northern area (Jeju-city) and the southern area (Seogwipo-city) of the island. Currently, Seogwipo-city is approximately 1.3°C warmer on average than Jeju-city in 2010.

Increased international travel, the transportation of eggs via the international trade in used tires, and transportation between countries are also important factors in the spread of VBDs together with the effects of climate change. Namely, international travelers and trade from endemic areas might serve as vehicles for further spread [6], [7], [8], [9].

Jeju Island has an international airport connected by direct flights to several nations in Asia and two seaports that receive vessels from many ports in East Asia. The geographical areas in which dengue transmission occurs have expanded to Taiwan in East Asia [9]. Therefore, arthropod vector species and their pathogens such as dengue fever vector and virus could be transmit from epidemic regions such as Southeast Asia to Jeju Island by globalization and, may survive in this area where they had not previously occurred due to warmer temperatures influenced by climate change [6], [7].

For these reasons, we have been studying the geographic distribution of medically important mosquito species such as *Aedes albopictus*, which transmit dengue fever, West Nile fever, yellow fever, St. Louis encephalitis, and chikungunya fever; *Culex pipiens*, which transmit West Nile fever and Japanese encephalitis; and *Anopheles sinensis*, which transmit vivax malaria, in several sites on Jeju Island since 2010. We also undertook a phylogenic analysis of *Ae. albopictus*, one of the important vectors of dengue fever, and study of the effects of climate change on the vectors and attempted to predict the future risk of transmission on Jeju Island.

## Materials and Methods

### Collection and identification of mosquitoes

**Figure 1:**
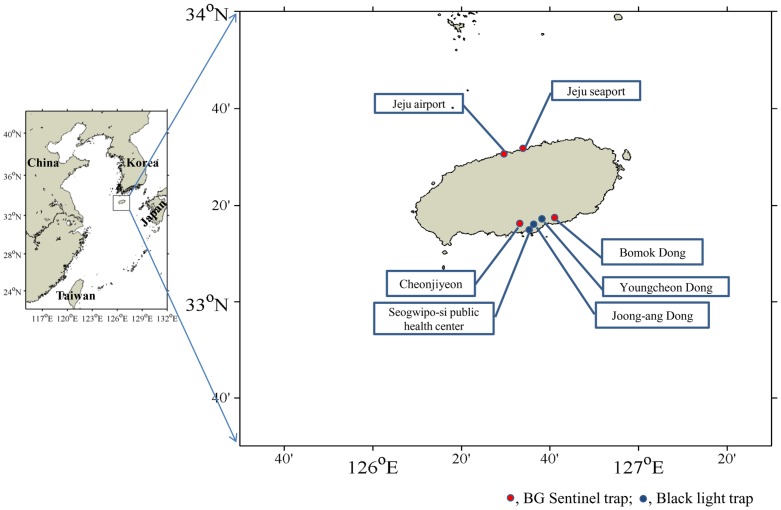
Mosquitoes sampling sites on Jeju Island.

From April 2010 to April 2011, we used 495 combined BG-Sentinel traps (Biogents, Regensburg, Germany) to capture *Aedes* species and black light traps (Shinyoung trade system, Gyeonggi-do, Korea) to capture *Culex* and *Anopheles* species on a weekly basis from seven locations across Jeju Island (Figure 1 and Table 1), and collection hours on a week is 48 hours. The Jeju-city sampling sites on the northern side of the island included the Jeju airport 1 and 2, and Jeju seaport 1 and 2. The Seogwipo-city sampling sites on the southern side of the island were Cheonjiyeon, Bomok dong, Seogwipo-city Public Health Center, Joong-ang dong, and Youngcheon dong. Collected mosquito vector were identified according to the manual of Korea Center for Disease Control and prevention [10]. *Ae. albopictus* isolates of Singapore, Vietnam, and Japan, and other region of Korea Peninsular were provided by Dr. N. Minakawa, Y. Higa, Y.S. Lee, and Y.S. Han respectively. This study was approved by Institutional Animal Care and Use Committee of Jeju National University. Sampling sites were not located at national parks or protected areas of land and this study was supported by the Korea Center for Disease Control. National quarantine stations in Jeju Island permitted the installation of traps around international air and sea ports.

**Table 1:** Mosquitoes sampling sites on Jeju Island.

Sampling site^a^	Latitude	Longitude	Remark
Jeju international airport 1, 2^b^	33° 30′ 26.74″N	126° 29′ 36.32″E	Jeju-city (urban area)
Jeju seaport 1, 2^c^	33° 31′ 13.94″N	126° 32′ 11.91″E	Jeju-city (urban area)
Cheonjiyeon	33° 14′ 38.76″N	126° 33′ 38.13″E	Seogwipo-city (forested area near the seaport)
Seogwipo-city Public Health Center	33° 15′ 10.95″N	126° 33′ 18.73″E	Seogwipo-city (urban area)
Bomok dong	33° 14′ 45.95″N	126° 36′ 2.44″E	Seogwipo-city (forested area)
Youngcheon dong	33° 16′ 7.34″N	126° 35′ 12.21″E	Seogwipo-city (urban area)
Joong-ang dong	33° 15′ 2.35″N	126° 33′ 54.32″E	Seogwipo-city (urban area)

a female adults were collected from April 2010 to April 2011;

b 0.3 km between airport 1 and 2;

c 0.4 km between seaport 1 and 2.

### Extraction of DNA from *Ae. albopictus* for phylogenetic analysis and viral RNA for virus detection using Real-Time Polymerase Chain Reaction

Phylogenetic analysis was performed on 109 *Ae. albopictus* specimens. Total mosquito DNA and viral RNA were extracted from the mosquitoes with a QlAamp Viral RNA Mini kit (Qiagen Inc., Mainz, Germany). Quantitative real-time PCR for flaviviruses yellow fever virus (YFV), dengue fever virus (DENV), Japanese encephalitis virus (JEV), and West Nile virus (WNV) was performed using commercial kit. An iNNOPLEX YFV/DENV Real-time PCR Typing kit (IP10132, iNtRON Biotechnology Inc. Seongnam-Si, Gyeonggi-do, Korea) was used to detect the YFV and DENV viruses while an iNNOPLEX JEV/WNV Real-time PCR Typing kit (IP10131, iNtRON Biotechnology Inc. Seongnam-Si, Gyeonggi-do, Korea) was used to detect JEV and West Nile virus WNV in a one-step real-time PCR [11]. The PCR mixture contained 8 µl of One-step RT-PCR premix, 7 µl of Detection Solution, and 5 µl of the RNA template in a total volume of 20 µl. PCR was performed under the following conditions: 30 min at 45°C, 10 min at 90°C, and 45 cycles of 15 s at 95°C and 30 s at 48°C.

### PCR and sequencing of *OBP*, *ND5*, and *COI* of *Ae. albopictus*

PCR for *OBP*, a 236-bp insect odorant-binding proteins (*OBP*), and two mitochondrial genes *ND5*, a 450-bp fragment of ND5 (NADH dehydrogenase subunit 5), and *COI*, a 507-bp fragment of COI (cytochrome oxidase subunit 1) were preformed respectively [12], [13], [14]. The two sets of primers used were: for *OBP*, Aalb OBP67 (5′-CGAGCCGTTCCAAATCGAAACCACCAGC-3′) and reverse primer Aalb OBP 67 (5′-CAAGGACCAATATGGAGAAGAGGATACTACAGCCG CTC-3′) [12]; for *ND5*, FORND5 (5′-TCCTTAGAATAAAATCCCGC-3′) and REVND5 (5′-GTTTCTGCTTTAGTTCATTCTTC-3′), and for *COI* FOR-COI (5′-GGAGGATTTGGAAATTGATTAGTTC-3′) and REV-COI (5′-CCCGGTAAAATTAAAATATAAACTTC-3′) [13], [14]. The PCR parameters were 5 min at 95°C, followed by 30 cycles of 30 s at 94°C, 45 s at 52°C, and 45 s at 72°C, with termination using a final extension step at 72°C for 10 min. The *OBP*, *ND5*, and *COI* PCR products were electrophoresed on a 1.2% agarose gel (Figure S1) and purified using a QIAEX II gel extraction kit (Qiagen Inc, Mainz, Germany) according to the manufacturer's instructions, and sequenced using a BigDye Terminator Cycle Sequencing kit (PerkinElmer Applied Biosystems, Warrington, UK) respectively.

### Phylogenetic analysis of *OBP*, *ND5*, and *COI*

The sequences of *OBP*, *ND5*, and *COI* were aligned using the multiple-alignment algorithm in the MegAlign program (Windows version 3.12e; DNASTAR, Madison, WI, USA) and the ClustalX program respectively [15]. Based on the aligned sequences, phylogenetic analyses were conducted in MEGA4 and phylogenetic trees were constructed by the neighbor-joining method. The bootstrap consensus tree inferred from 1000 replicates is taken to represent the evolutionary history of the taxa analyzed [16].

### Statistical analysis for the distribution of mosquitoes, mean temperature, and amount of precipitation

We wish to study the relationship between the numbers of types *Ae. albopictus*, *Cx. pipiens*, and *An. sinensis* mosquitoes from particular sites on Jeju Island and associated variables. We considered the Poisson regression, a type of generalized linear. This model is useful when the responsible variable is discrete and countable [17]. The number of *Ae. albopictus*, *Cx. pipiens*, and *An. sinensis* mosquitoes are considered as a response variable. The associated predictor variables include site specific monthly mean temperature and precipitation. The maximum likelihood estimates of the poisson regression parameters were obtained through SAS version 9.1.

## Results

### Distribution of mosquitoes species in Jeju Island from April 2010 to April 2011

We collected medically important mosquito species such as *Ae. albopictus*, which can transmit dengue fever, *Cx. pipiens*, which can transmit West Nile fever, and *An. sinensis*, which can transmit malaria, on Jeju Island from April 2010 to April 2011. The total female abundance of *Ae. albopictus*, *Cx. pipiens*, and *An. sinensis* is 1225, 9929 and 63 respectively. *Aedes aegypti* is another important vector of dengue but is has not been found on Jeju Island, and was not collected in our study.

**Figure 2:**
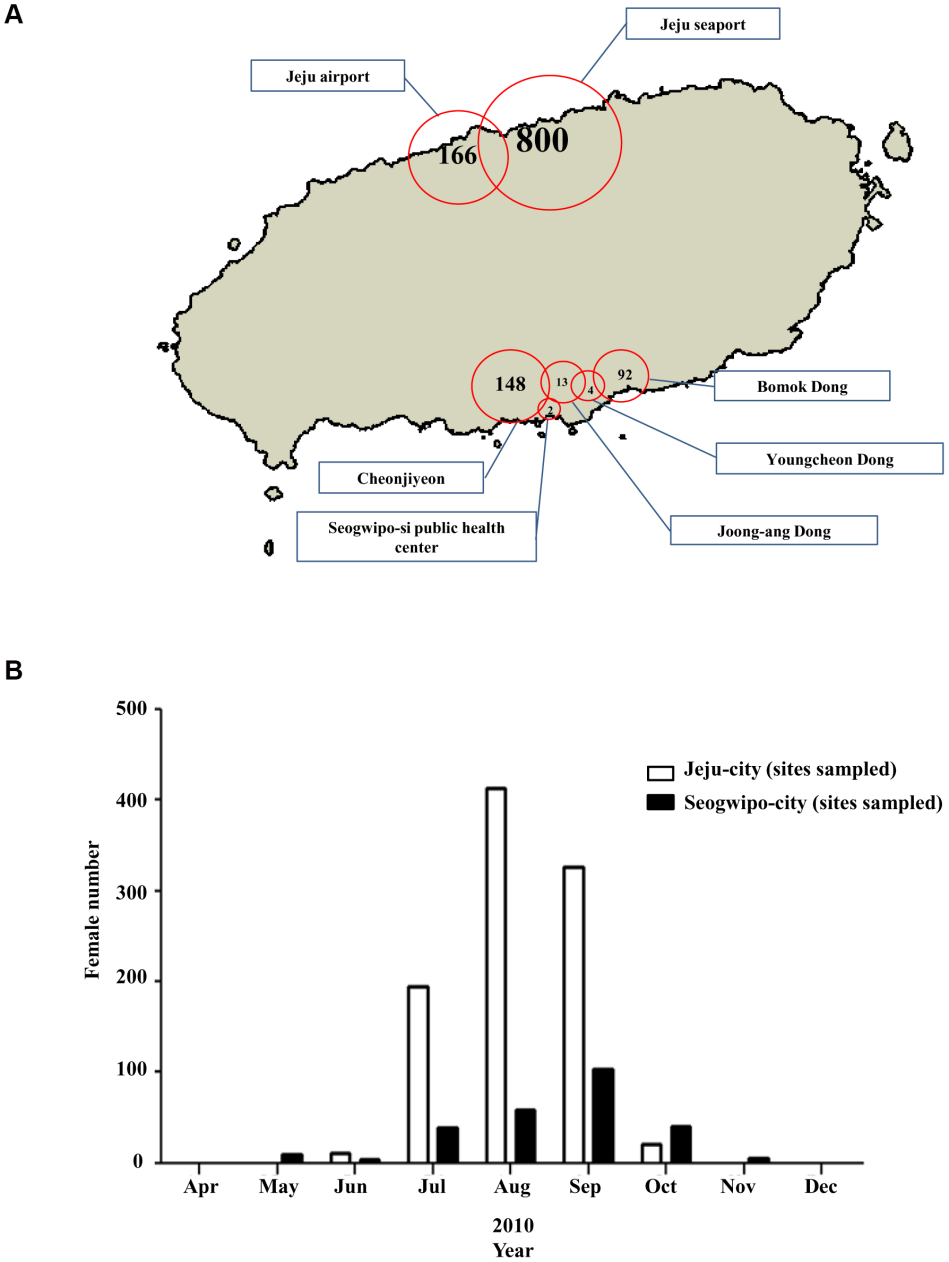
Total abundance of females of *Ae.* *albopictus*. Total abundance of females of *Ae. albopictus* at the sampling sites on Jeju Island (A) and monthly abundances of females of *Ae. albopictus* to the northern and southern areas of Jeju Island (B).

In Jeju-city, *Ae. albopictus* were collected mainly at influx sites such as the Jeju seaport and Jeju international airport in Jeju-city, and at Cheonjiyeon, located near the Seogwipo seaport, and Bomok Dong, a forested area (Figure 2A), in Seogwipo-city. *Ae. albopictus* were more prevalent in Jeju-city than in Seogwipo-city. They were collected in Seogwipo-city in the seven months from May through November and in Jeju-city for the five months from June through October (Figure 2B).

**Figure 3:**
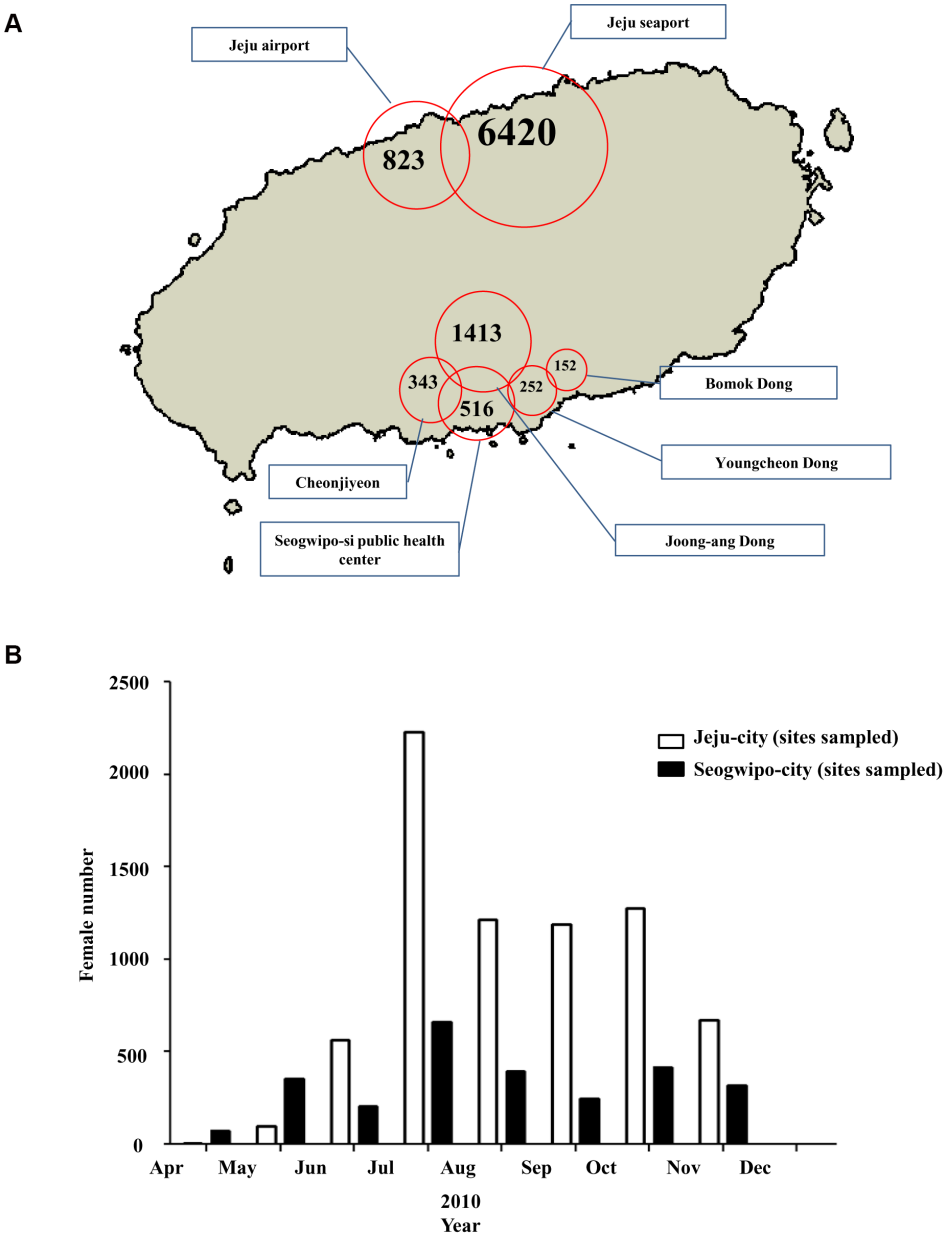
Total abundance of females of *Cx.* *pipiens*. Total abundance of females of *Cx. pipiens* at the sampling sites on Jeju Island (A) and monthly abundances of females of *Cx. pipiens* to the northern and southern areas of Jeju Island (B).

The distribution of *Cx. pipiens* was similar to that of *Ae. albopictus*, although in Seogwipo- city, they were not collected at forested areas but rather at urban areas such as Joong-ang dong and the Seogwipo-city Public Health Center (Figure 3A). The monthly data for *Cx. pipiens* were similar to those of *Ae. albopictus*, but *Cx. pipiens* were collected over a longer period. In Jeju-city, *Cx. pipiens* appeared in May. The greatest number of insects were collected in July and they disappeared in November. In Seogwipo-city, *Cx. pipiens* appeared in April, the numbers fluctuated from May to November, and they disappeared in December (Figure 3B).

**Figure 4:**
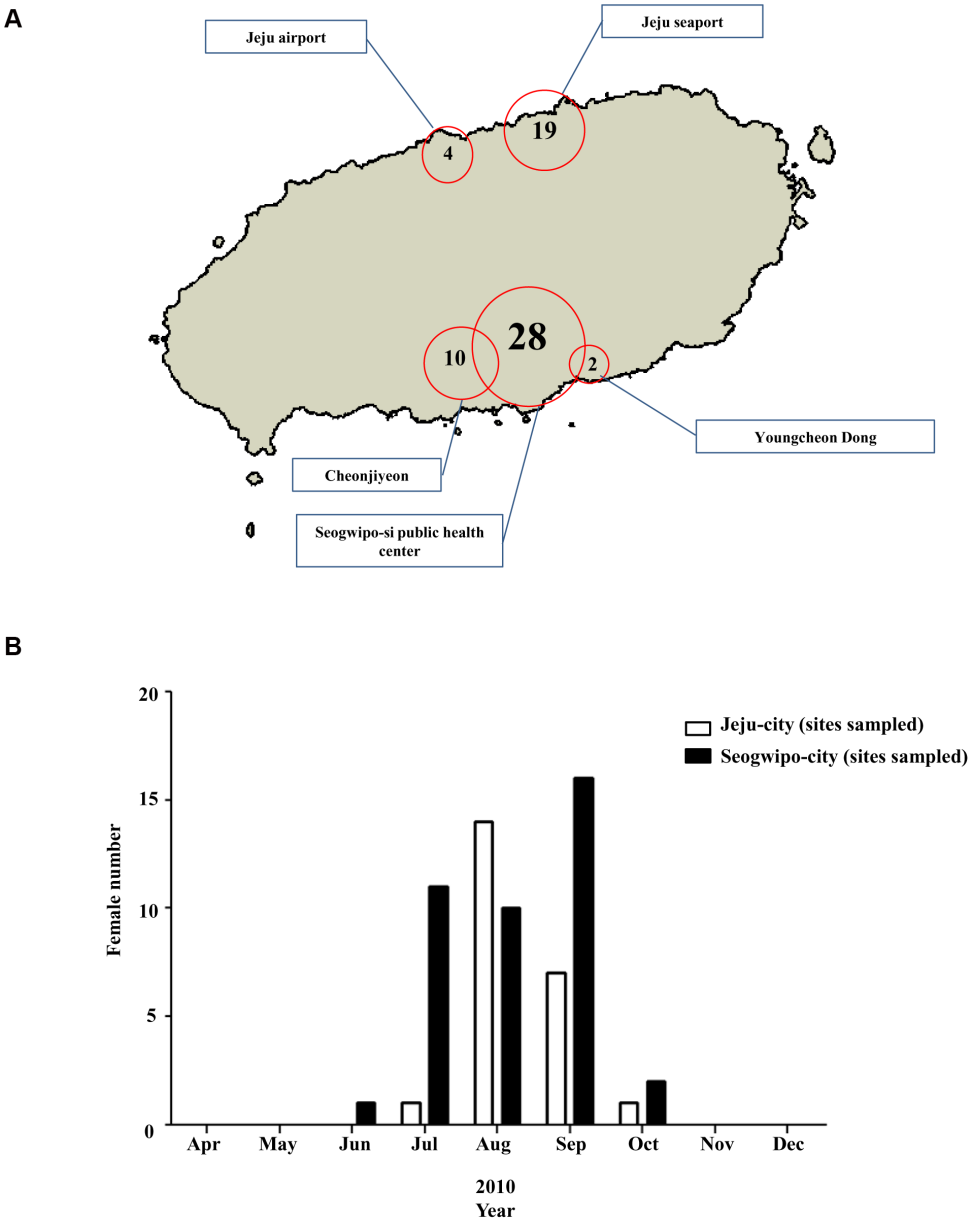
Total abundance of females of *An.* *sinensis* Total abundance of females of *An. sinensis* at the sampling sites on Jeju Island (A) and monthly abundances of females of *An. sinensis* to the northern and southern areas of Jeju Island (B).

Unlike *Ae. albopictus* and *Cx. pipiens*, *An. sinensis* were collected at influx areas such as Jeju seaport and international airport and in an urban area such as Seogwipo-city Public Health Center. This was an unexpected result because *An. sinensis* is usually found in forested areas and not urban areas (Figure 4A). In Jeju-city, *An. sinensis* appeared in July and its number increased markedly for one month and then decreased from September to October. However, its total count was lower than that of the other mosquito species. In Seogwipo-city, *An. sinensis* appeared in June; a month earlier than in Jeju-city, reached its highest number in September, and disappeared in November (Figure 4B).

We also tried to isolate viruses from collected mosquito vectors using real-time PCR. But, virus pathogen did not exist in mosquito vectors. Until now, mosquito vectors hibernate in during the colder winter season, usually from December to February, in Jeju Island, and as a result virus did not exist in these mosquitoes.

### Phylogenetic analysis of *Ae. albopictus* isolates in Jeju Island using *OBP* and two mitochondrial genes, *ND5* and *COI* respectively

**Figure 5:**
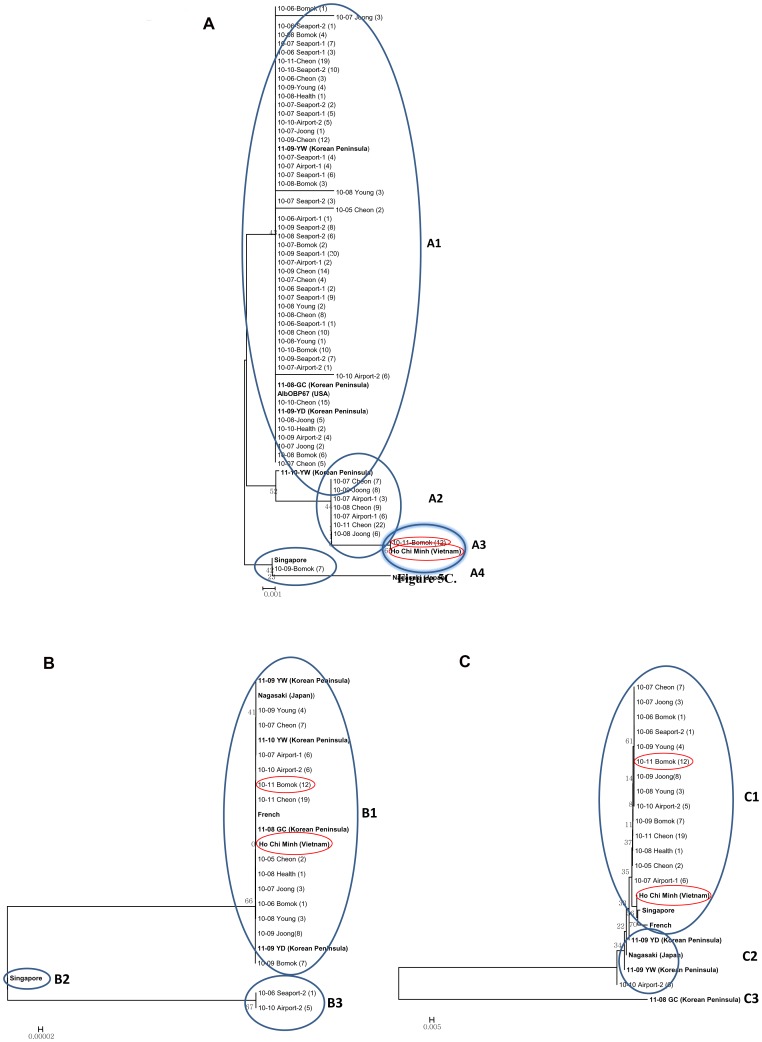
Phylogenetic tree constructed based on the partial atypical *OBP* (A), *ND5* (B), and *COI* (C). The tree was constructed using the neighbor-joining method in MEGA4. YW (Youngwol), collected in the Southwest of the Korean Peninsula, GC (Gimcheon), collected in Southeast of the Korean Peninsula, YD (Yeongdo), collected in the south of the Korean Peninsula, Ho Chi Minh Vietnam, Singapore, Nagasaki Japan, AlbOBP67 USA, and French isolates are in bold. Each sample was identified as year-month-collection site. The *OBP* and *ND5* sequences of 10-11-Bomok, which was collected in November 2010, and in a forest area of Seogwipo-city, were the same as those in the collect of Ho Chi Minh Vietnam.

**Figure 6:**
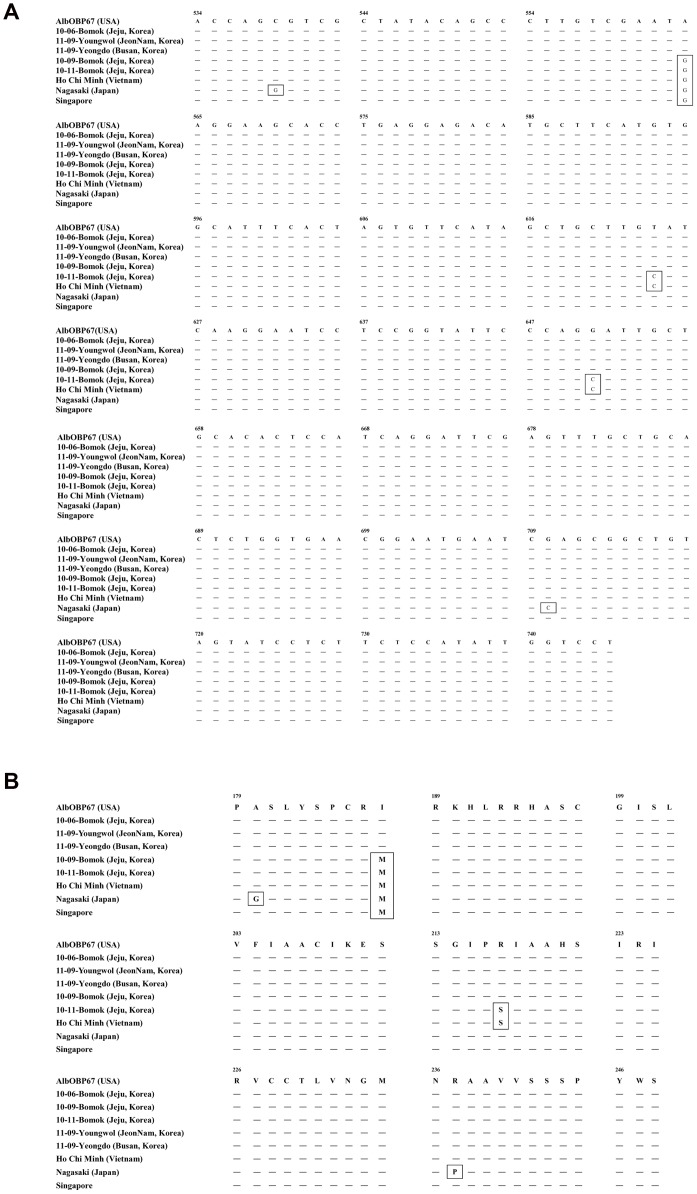
OBP's nucleotide and amino acid sequences. Alignment of OBP's nucleotide sequences (A) and OBP's amino acid sequences (B) about AlbOBP67 USA, Ho Chi Minh Vietnam, Nagasaki Japan, Singapore, 10-06-Bomok, 11-09-Youngwol (YW), 11-09-Yeongdo (YD), 10-09-Bomok, and 10-11-Bomok isolates.

We performed a phylogenetic analysis using *OBP* and two mitochondria genes, *ND5* and *COI*. OBPs play a critical role in mediating mosquito behaviors, and this gene is used in the phylogenetic analysis for *Ae. albopictus* [12]. *OBP* was amplified and sequenced for 109 *Ae. albopictus* obtained on Jeju Island, and these sequences were compared with those of Korean peninsula isolates and isolates from Ho Chi Minh, Vietnam; Singapore; Nagasaki, Japan; and USA (AlbOBP67). *Ae. albopictus* of Jeju Island were separated into four groups (A1 to A4). On October 2010, the 10-11-Bomok isolate from Bomok dong in the southern area of island had the same sequence of *Ae. albopictus* of Ho Chi Minh. It differed from specimens from Nagasaki (A4), even though Nagasaki is located at the same latitude as Jeju Island and is closer than Ho Chi Minh (Figure 5A, Figure 6A, 6B).

We also performed phylogenetic analysis using two mitochondria genes *ND5* and *COI*, and used these to create an *OBP* phylogenetic tree [13], [14]. The *ND5* phylogenetic tree showed that the isolates from Bomok Dong (10-11-Bomok), Ho Chi Minh, and Nagasaki were in the same group (B1) (Figure 5B). The *COI* tree showed that the isolates from Bomok Dong (10-11-Bomok) were closer to those from Ho Chi Minh and Singapore (C1) than to the isolate from Nagasaki (C2) (Figure 5C). These phylogenetic trees, especially the *OBP* phylogenetic tree, suggest that the mosquito vector entering through sea and airports and surviving in the southern portion of Jeju Island may originate from Vietnam, an endemic area of dengue [3].

### Mean Temperature in Jeju-city and Seogwipo-city from 1970 to 2011

**Figure 7:**
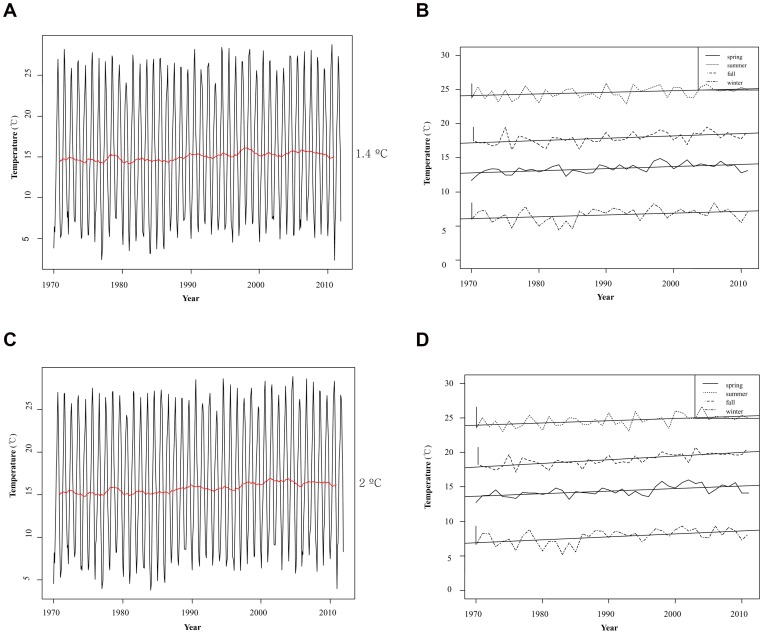
Mean temperature and trend of seasonal temperature of Jeju Isalnd. Mean temperature and trend of seasonal temperature of Jeju-city (A and B), Seogwipo-city (C and D), were recorded from 1970 to 2011.

Mean temperature in Jeju-city and Seogwipo-city were recorded by the National Institute of Meteorological Research from 1970 to 2011. During this time, the mean temperature of Jeju-city increased by 1.4°C, from 14.8°C to 16.2°C (Figure 7A), and the mean temperature of Seogwipo-city increased by 2°C, from 15.5°C to 17.5°C (Figure 7C).

### Relationships between the distribution of mosquitoes, mean temperature, and amount of precipitation

We investigated the relationships between the distribution of mosquitoes and climate factors such as site-specific monthly mean temperature and precipitation.

Table 2 shows that the density of each mosquito species has high correlation with site-specific mean temperature and amount of precipitation (*p*<0.001). The mean temperature and amount of precipitation were higher in Seogwipo-city than in Jeju-city. These results suggest that the mean temperature and amount of precipitation are important climate factor affecting the distribution of mosquitoes in Jeju Island, and Seogwipo-city is more vulnerable area than Jeju-city.

**Table 2:** Results of generalized linear models (Poisson Regression Model) to test three explanatory variables of the density of each mosquito species.

	Site	Mean temperature^a^	Amount of precipitation^b^
*Ae.albopictus*	*p*<0.0001	*p*<0.0001	*p*<0.0001
*Cx. pipiens*	*p*<0.0001	*p*<0.0001	*p*<0.0001
*An.sinensis*	*p*<0.0001	*p*<0.0001	*p*<0.0001

a Mean temperature = Monthly mean temperature for each site;

b Amount of precipitation = Monthly Amount of precipitation for each site.

## Discussion

In the past 100 years, the mean surface temperature has increased by 0.3–0.8°C across the Asian continent and is projected to increase by 0.4–4.5°C by 2070 [1], [18]. Jeju Island is a volcanic island located between 126°00′ and 126°58′ E longitude and 33°06′ and 34°00′N latitude; it is 73 km wide and 41 km long with a total area of 1,847 km^2^ [19]. This island is located at the southern end of the Korean Peninsula which is the hottest area and is classified as a subtropical weather zone [5]. Thus, the island would be affected more by climate change than other regions of South Korea. The Island's warm climate, with an average temperature 16.1°C (north) to 17.4°C (south), and moderate precipitation, averaging 1,388 mm (north) to 1,782 mm (south), provide a favorable environment for the growth and spreading of vectors [19].

From 1970 to 2011, the annual mean temperature of Jeju Island increased more compared with other regions in the Korean Peninsula. The temperature increased by 1.4°C in Jeju-city (nouthern areas) and by 2.0°C in Seogwipo-city (southern areas); Seogwipo-city has a 1–2°C higher temperature than Jeju-city (Figure 7A, 7C).

VBDs are one of the major contributors to the burden of disease. Mosquito species are responsible for the transmission of most VBDs and are highly sensitive to climatic conditions such as temperature and rainfall [1], [9], [20]. Mosquito species are very sensitive to temperature changes in both the immature stages in the aquatic environment and as adults. The larvae mature faster in warmer water, and there is thus a greater capacity to produce more offspring during the transmission period [2], [6], [21]. In warmer climates, adult female mosquitoes digest blood faster and feed more frequently, thus increasing transmission intensity [22]. The development rates of vector-borne pathogens also are strongly temperature dependent [4]. Therefore, climate change affects the life of vectors and the development rates of vector-borne pathogens and the risk of VBDs transmission will be increase. Our statistical analysis showed significant associations between the mean temperature, amount of precipitation, and density of mosquito vectors in Jeju Island (Table 2).

Climate change also causes mosquitoes to appear earlier and disappear later than previously, and some species may be able to survive during the colder winter months. In Seogwipo-city, mosquito vectors started to appear from April, which was earlier than in Jeju-city and other regions in the Korean Peninsula such as Yeongnam (Southeast areas of Korean Peninsula) and Honam (Southwest areas of Korean Peninsula), and survived longer to November (Figure 2B, 3B and 4B).

*Ae. albopictus* is a vector for dengue, the geographic distribution of *Ae. albopictus*, and dengue transmission has been extending steadily toward East Asia [8], [9], [18], [23] and is expected to expand to Korea, especially Jeju-Island and Japan, by the effects of increased international travel and climate change.

The results of virus detection in mosquito vectors using Real-Time PCR show *Ae. albopictus*, which transmit dengue fever virus, and *Cx. pipiens*, which transmit West Nile virus, in Jeju Island did not have viral pathogens, but the *OBP* phylogenetic tree of *Ae. albopictus* show the inflow of this mosquito from Vietnam to Jeju Island (Figure 5A).

Hales S *et al.*, showed that in 1990, almost 30% of the world population, totaling 1.5 billion people, lived in regions where the estimated risk of dengue transmission was greater than 50%. With population and climate change projections for 2085, they estimate that about 5–6 billion people (50–60% of the projected global population) would be at risk of dengue transmission [23]. Therefore, our results suggest that virus-carrying mosquito vectors may inflow through seaports and international airports from VBD outbreak areas (endemic areas), and may be able to survive all year by the rising temperatures in Jeju Island and newly emerging VBDs, especially dengue, could outbreak in Jeju Island one day by the effects of global climate change and globalization such as increased international travel, trade, and transportation [8], [23], [24].

That is, if climate change continues in Jeju Island may no longer be a region safe from VBDs such as dengue. Geographic and climate factors such as mean temperature and amount of precipitation temperature on Jeju Island should be monitored to identify new emerging indigenous VBDs in Jeju Island for prevention of newly emerging VBDs in East Asia.

## Supporting Information

Figure S1 **Amplification of** ***OBP*** **,** ***ND5*** **, and** ***COI*** **.** Lanes M, marker DNA (25- and 100-bp mixed DNA ladder); 1, Sample 1; 2, Sample 2; 3, Sample 3.
(TIF)
