# Supplementary figures and images for: The Effects of Climate Change and Globalization on Mosquito Vectors: Evidence from Jeju Island, South Korea on the Potential for Asian Tiger Mosquito (Aedes albopictus) Influxes and Survival from Vietnam Rather Than Japan
Source: PLoS One. 2013 Jul 24;8(7):e68512. doi: 10.1371/journal.pone.0068512 (PMC3722226; doi:10.1371/journal.pone.0068512)

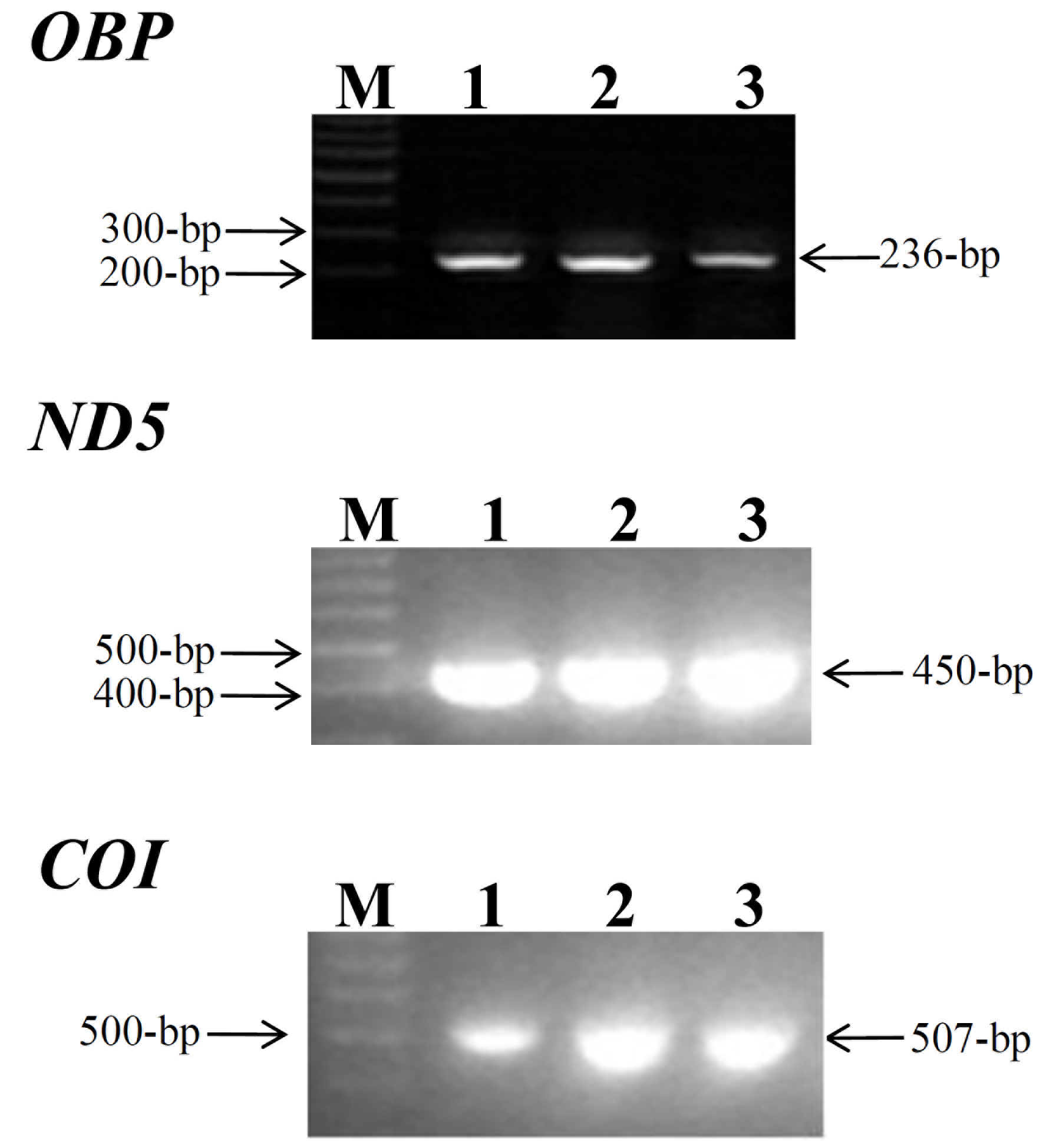

Supplement: Figure S1 — Amplification of OBP , ND5 , and COI . Lanes M, marker DNA (25- and 100-bp mixed DNA ladder); 1, Sample 1; 2, Sample 2; 3, Sample 3. (TIF) [file pone.0068512.s001.tif]
